# Interleukin-36 family dysregulation drives joint inflammation and therapy response in psoriatic arthritis

**DOI:** 10.1093/rheumatology/kez358

**Published:** 2019-09-03

**Authors:** Marie-Astrid Boutet, Alessandra Nerviani, Gloria Lliso-Ribera, Davide Lucchesi, Edoardo Prediletto, Giulia Maria Ghirardi, Katriona Goldmann, Myles Lewis, Costantino Pitzalis

**Affiliations:** Centre for Experimental Medicine & Rheumatology, William Harvey Research Institute and Barts and The London School of Medicine and Dentistry, Queen Mary University of London, London, UK

**Keywords:** psoriatic arthritis, rheumatoid arthritis, synovitis, inflammation, cytokines, early arthritis, interleukin-36

## Abstract

**Objectives:**

IL-36 agonists are pro-inflammatory cytokines involved in the pathogenesis of psoriasis. However, their role in the pathogenesis of arthritis and treatment response to DMARDs in PsA remains uncertain. Therefore, we investigated the IL-36 axis in the synovium of early, treatment-naïve PsA, and for comparison RA patients, pre- and post-DMARDs therapy.

**Methods:**

Synovial tissues were collected by US-guided biopsy from patients with early, treatment-naïve PsA and RA at baseline and 6 months after DMARDs therapy. IL-36 family members were investigated in synovium by RNA sequencing and immunohistochemistry, and expression levels correlated with DMARDs treatment response *ex vivo*. Additionally, DMARDs effects on IL-36 were investigated *in vitro* in fibroblast-like synoviocytes.

**Results:**

PsA synovium displayed a reduced expression of IL-36 antagonists, while IL-36 agonists were comparable between PsA and RA. Additionally, neutrophil-related molecules, which drive a higher activation of the IL-36 pathway, were upregulated in PsA compared with RA. At baseline, the synovial expression of IL-36α was significantly higher in PsA non-responders to DMARDs treatment, with the differential expression being sustained at 6 months post-treatment. *In vitro*, primary PsA-derived fibroblasts were more responsive to IL-36 stimulation compared with RA and, importantly, DMARDs treatment increased IL-36 expression in PsA fibroblasts.

**Conclusion:**

The impaired balance between IL-36 agonists–antagonists described herein for the first time in PsA synovium and the decreased sensitivity to DMARDs *in vitro* may explain the apparent lower efficacy of DMARDs in PsA compared with RA. Exogenous replacement of IL-36 antagonists may be a novel promising therapeutic target for PsA patients.

**Rheumatology key messages**

- A high ratio between IL-36 agonists (pro-inflammatory) and antagonists (anti-inflammatory) characterizes PsA synovial tissue.
- PsA-derived synovial fibroblasts are more responsive to IL-36 stimulation compared with RA.
- Higher expression of synovial IL-36 predicts inadequate response to DMARDs in PsA but not RA.

## Introduction

PsA is a chronic seronegative SpA affecting up to 30% of patients with skin psoriasis and characterized by the presence of spondylitis, enthesitis and peripheral arthritis [1]. The use of biologic agents such as TNF-α blockers and those targeting the IL-23/IL-17 axis has improved the disease outcome of PsA patients [2]. However, up to 30–40% of patients [3], especially those suffering from peripheral articular inflammation, do not respond adequately to the available treatments, hence the need for new therapeutic targets.

The IL-36 family, part of the larger IL-1 cytokine family, includes three agonists, IL-36α, IL-36β and IL-36γ, and two inhibitors, IL-36RA and IL-38. IL-36RA specifically antagonizes IL-36α, IL-36β and IL-36γ by binding IL-1Rrp2 with high affinity [4], thus preventing the recruitment of the IL-1RAcP subunit and reducing the downstream activation of nuclear factor (NF)-κB or MAPK pathways [5]. IL-38 is a broader inhibitor able to antagonize additionally to IL-36 cytokines, Toll-Like Receptor and the IL-1-mediated signalling pathway [6–8]. Like IL-1, IL-36 cytokines need to be processed to be fully active and neutrophils proteases have been identified as the main regulators of IL-36 axis activation [9–13]. The pathogenic role of IL-36 in the initiation and maintenance of inflammation in skin psoriasis has been extensively proven [14–16]. In mouse models of psoriasis, IL-36α, in crosstalk with IL-1 and Th17, promotes neutrophil recruitment and chemokines production [17], while mice lacking IL-1R1 and IL-36α are almost disease-free [18]. In humans, the absence of the IL-36 inhibitor IL-36RA causes acute generalized pustular psoriasis [19], and IL-36R inhibitors are currently on trial for treating psoriasis after successful studies in mice [20, 21].

IL-36 cytokines are also detected in the synovium of RA patients and can stimulate the production of pro-inflammatory mediators by synovial fibroblasts [22–24]; however, it seems that the IL-36R blockade has no beneficial effects in several mouse models of arthritis, implying a prevalent pathogenic role of IL-36 in skin rather than joint disease [25–27]. The expression and functions of IL-36 in PsA synovium have hardly been defined, and only one study has so far demonstrated that IL-36α is expressed within the PsA synovium at a similar level compared to the RA synovium in established disease [28].

However, to our knowledge, there are no studies systematically investigating the IL-36 family (agonists/antagonists) in treatment-naïve early arthritis synovium. Since disease pathology and cytokine expression can be modified by DMARDs therapy, in this study we report for the first time the expression pattern of IL-36 cytokines pre/post-DMARDs therapy in early treatment-naïve PsA synovium in comparison with RA, and their modulation by DMARDs treatment of Fibroblast-Like Synoviocytes (FLS) *in vitro*.

## Methods

### Synovial biopsies and plasma samples

Synovial tissue (ST) and plasma samples were collected from early (<12 months of symptoms) treatment-naïve RA and PsA patients enrolled into the Pathobiology of Early Arthritis Cohort at Bart’s Health National Health Service Trust [29]. Patients underwent US-guided needle synovial biopsy of an actively inflamed joint prior to and 6 months after receiving DMARDs [30]. A summary of patients’ characteristics is presented in supplementary Table S1, available at *Rheumatology* online. ST fragments were embedded in paraffin for histological characterization or preserved in RNAlater (Ambion, Invitrogen, Carlsbad, CA, USA) for gene expression analysis. All RA patients fulfilled the 2010 ACR/EULAR criteria [31], while PsA was diagnosed based on clinical grounds. All patients gave written informed consent. The study was approved by the National Research Ethics Service Committee London Dulwich (REC 05/Q0703/198).

### Whole ST RNA extraction and sequencing

Total RNA was extracted from the ST using a Trizol/Chloroform method. Bulk RNA sequencing was performed on an Illumina HiSeq2500 platform (Illumina Inc., San Diego, CA, USA). Raw data were quality-controlled using FastQC, trimmed or removed with Cutadapt. Transcript abundance was derived from paired sample FASTQ files over GENCODE-v24/GRCh38 transcripts using Kallisto-v0.43.0. Normalization and analysis of regularized log expression read counts were performed using DESeq2-v1.22.1 package in R-v3.5.2 statistics. RNA sequencing data have been uploaded to ArrayExpress and are accessible via accession E-MTAB-6141.

### Immunohistochemistry and multiple immunofluorescent labelling

Sequential 3-µm-thick sections of ST underwent haematoxylin and eosin and immunohistochemical staining to determine the level of inflammation and the degree of cellular infiltration by B cells (CD20+, Dako, Agilent Technologies, Santa Clara, CA, USA), T cells (CD3+, Dako), plasma cells (CD138+, Dako), lining/sublining macrophages (CD68+, Dako) and fibroblasts (TE7+, Merck, Darmstadt, Germany). Synovial samples were categorized into three pathotypes (pauci-immune, diffuse or follicular) following semi-quantitative scoring by two independent observers [32]. STs were also stained for IL-36α (Sigma-Aldrich, St Louis, MO, USA), IL-36β (Sigma-Aldrich), IL-36γ (Novus Biologicals, Centennial, CO, USA), IL-36Ra (R&D Systems, Minneapolis, MN, USA), IL-38 (Thermo Fisher Scientific, Waltham, MA, USA), IL-36R (Novus Biologicals), Neutrophil Elastase (Novus Biologicals), Cathepsin G (Abcam, Cambridge, UK) and Cathepsin S (Abcam) as previously described [22, 33]. Matching isotype controls [rabbit and mouse IgG2b (Dako), mouse IgG1 (Abcam) and IgG2a (Biolegend, San Diego, CA, USA)] were used to confirm the specificity of the primary antibodies. Slides were counterstained with haematoxylin and mounted with Distyrene Plasticizer Xylene mountant (Sigma-Aldrich). For double fluorescent labelling, sections were incubated simultaneously with IL-36α together with CD68, CD138, CD3, CD20 or TE7. Alexa-Fluor 488- or 594-conjugated goat anti-rabbit or -mouse (Invitrogen, Carlsbad, CA, USA; Thermo Fisher Scientific) were used as secondary antibodies. Slides were counterstained with 40, 6-diamidino-2-phenylindole (Invitrogen, Thermo Fisher Scientific) and mounted with ProLong Antifade mountant (Thermo Fisher Scientific). Triple immunofluorescence staining was performed using a tyramide signal amplification protocol in order to evaluate the co-expression of IL-36α, IL-36Ra and IL-36R. Briefly, after incubation with each primary antibody followed by the appropriate EnVision+ system horseradish peroxidase (Dako) anti-mouse or anti-rabbit for 30 min, the Alexa-Fluor 488-, Alexa-Fluor 555- or Cy5-conjugated tyramide reagents (Invitrogen, Thermo Fisher Scientific) were added per manufacturer instructions. Each primary antibody complex was stripped before the subsequent by microwaving the slides for 15 min at low power in citrate retrieval solution (pH 6, Dako). Nuclei were counterstained with 6-diamidino-2-phenylindole and slides mounted with ProLong Antifade mountant. All sections were visualized with a BX61 microscope (Olympus, Tokyo, Japan) or the digital slide scanner Nanozoomer S60 (Hamamatsu Photonics, Japan). Details of antibodies characteristics and concentrations used are presented in supplementary Table S2, available at *Rheumatology* online. Quantitative digital image analyses were performed using ImageJ software (National Institutes of Health, Bethesda, MD, USA).

### FLS isolation and stimulation

FLS were isolated from RA/PsA ST obtained by either needle biopsy or joint replacement (Research Tissue Biobank, REC 17/WS/0172 approved by the West of Scotland REC 4 Research Ethics) as previously described [22]. Cells were either stimulated with rhIL-36α and/or rhIL-36RA (R&D Systems, Minneapolis, MN, USA) or with IL-1β (25 ng/ml, R&D Systems) and/or TNF-α (5 ng/ml or 25 ng/ml, R&D Systems) and with MTX or sulfapyridine (SP) (1 mM, Sigma-Aldrich, St Louis, MO, USA) as specified in the figures legends. Dimethyl sulfoxide (DMSO) alone was used as control for MTX and SP stimulations. For immunocytofluorescence staining, FLS were seeded on glass slides and fixed before proceeding to the staining.

### Western blot

FLS were lysed in radioimmunoprecipitation assay buffer supplemented with protease and phosphatase inhibitors (Sigma-Aldrich, St Louis, MO, USA). Samples were loaded on precast Protean gels (Bio-Rad, Hercules, CA, USA). Primary antibodies [anti-human NF-κBp65 (Santa Cruz, Dallas, TX, USA), phosphoNF-κBp65 (Cell Signalling, Denvers, MA, USA), Actin (Sigma-Aldrich)] and corresponding secondary antibodies (Santa Cruz) were used to detect proteins of interest.

### ELISA assays

IL-36α, IL-38 (R&D Systems, St Louis, MO, USA) and IL-36RA (MyBioSource, San Diego, CA, USA) levels in plasma and IL-8 (Thermo Fisher Scientific, Waltham, MA, USA) and IL-6 (Biolegend, San Diego, CA, USA) concentration in cells supernatants were determined according to the manufacturer’s instructions.

### Primary cells RNA extraction, RT and quantitative PCR analysis

RNA from primary cells was recovered using Direct-zol RNA-MiniPrep kit (ZymoResearch, Irvine, CA, USA) and cDNA prepared using Superscript IV First-Strand Synthesis System (Invitrogen, Carlsbad, CA, USA; Thermo Fisher Scientific, Waltham, MA, USA). Gene expression was quantified using TaqMan probes/buffers and acquired on a 7900HT Fast-Real Time System (all Thermo Fisher Scientific). Genes were normalized against the expression of Glyceraldehyde-3-Phosphate Dehydrogenase.

### Statistical analysis

Differences in quantitative variables were evaluated by the Mann–Whitney *U* test (two groups, <30), unpaired Student’s *t* test (two groups, >30) or Kruskal–Wallis with Dunn’s post-test (multiple groups). Fisher’s exact test (<30) or χ^2^ (>30) was used to evaluate associations of qualitative variables. Correlations were evaluated by Spearman’s bivariate analysis. Statistical analyses were performed using GraphPad Prism-v6 software (Graphpad, San Diego, CA, USA). *P*-values <0.05 were considered significant.

## Results

### IL-36 antagonists IL-36RA and IL-38 are expressed at lower levels in PsA *vs* RA synovium

**Fig. 1:**
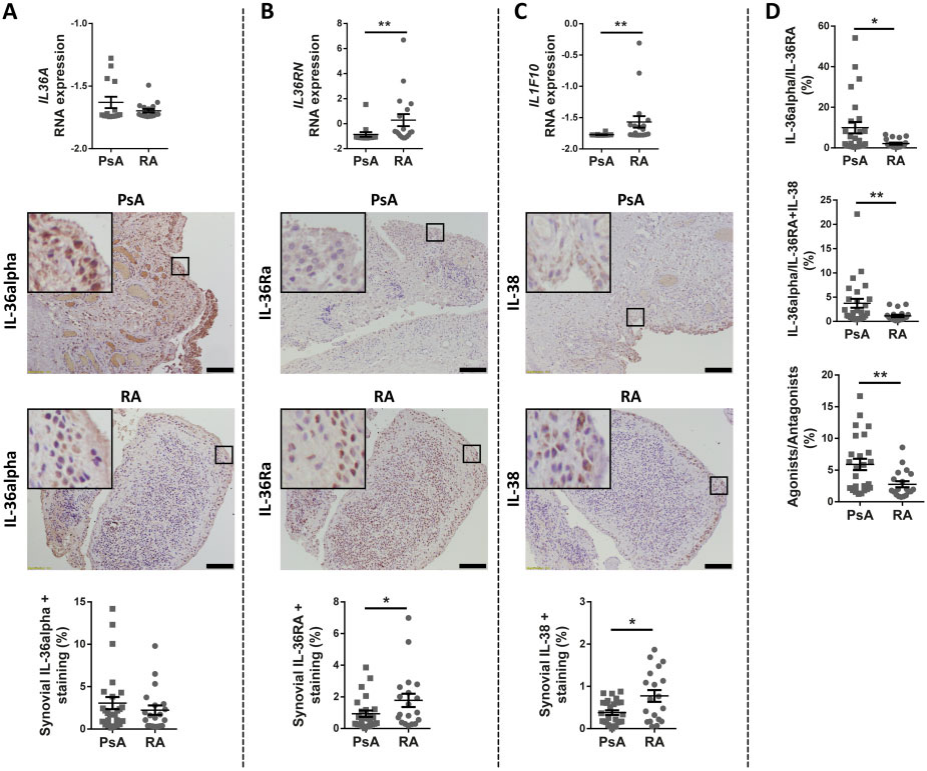
Expression of IL-36α, IL-36RA and IL-38 in synovium from early DMARDs-naïve PsA and RA patients mRNA expression of IL-36A (**A**), IL-36RN (**B**) and IL-1F10 (**C**) in synovium of PsA and RA patients was assessed by RNA sequencing; *n*=14 PsA and 18 RA. Sections of human PsA and RA synovium were stained for IL-36α (A), IL-36RA (B) and IL-38 (C). Representative images are shown. Scale bar=100 μm. Enlarged images correspond to the respective boxed areas. Digital image analysis was performed on all PsA (*n*=27) and RA (*n*=19) synovium sections. IL-36α+ (A), IL-36RA+ (B) and IL-38+ (C) surfaces were determined using ImageJ software and are presented as % of the synovium surface. (**D**) Ratios between agonist (IL-36α) and antagonists (IL-36RA and IL-38) expressions are shown. (A–D) Results are presented as mean (s.e.m). *P < 0.05, **P < 0.01 as assessed by Mann–Whitney *U* test.

In keeping with previously published data [28], we detected comparable synovial levels of the agonist IL-36α in PsA and RA tissues, both at RNA and protein level (Fig. 1A). In contrast, we found an impaired expression of IL-36 antagonists IL-36RA and IL-38 in PsA synovium compared with RA, with significantly lower expression confirmed at both RNA and protein level by immunohistochemistry (Fig. 1B and C). IL-36α is here shown as representative of the IL-36 agonists, but the same trend was observed also for IL-36β and IL-36γ (supplementary Fig. S1A and B, available at *Rheumatology* online). In PsA ST, the ratio between the IL-36 agonists and inhibitors, both the selective IL-36RA and the non-specific IL-38, mirrored the same relationship observed in psoriatic skin and was significantly higher compared with RA, further suggesting a deficit of expression of the IL-36 antagonists in PsA (Fig. 1D and supplementary Fig. S1C, available at *Rheumatology* online).

### IL-36-activating neutrophil proteases and neutrophil signatures are increased in PsA *vs* RA synovium

**Fig. 2:**
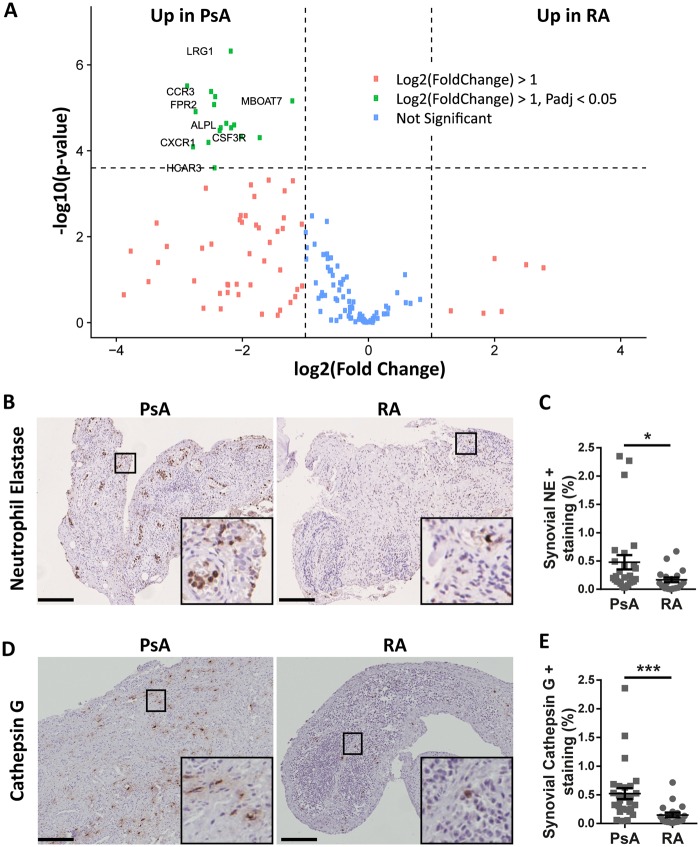
PsA synovium is characterized by a strong neutrophil signature (**A**) Volcano plot representation of differential expression of neutrophils-associated genes in PsA and RA synovium. Green points mark the genes with significantly increased expression respectively in PsA compared with RA synovium. The *x*-axis shows log2-fold changes in expression and the *y*-axis the log odds of a gene being differentially expressed. (**B**, **D**) Sections of human PsA and RA synovium were stained for NE (B) and Cathepsin G (D). Representative images are shown. Scale bar = 100 μm. Enlarged images correspond to the respective boxed areas. (**C**, **E**) Digital image analysis was performed on all PsA (*n*=27) and RA (*n*=19) synovium sections. NE (C) and Cathepsin G (E) positive staining surfaces were determined using ImageJ software and are presented as % of the synovium surface. Results are presented as mean (s.e.m). *P < 0.05, ***P < 0.001 as assessed by Mann–Whitney *U* test. NE: Neutrophil elastase.

To become functional, IL-36 cytokines must be cleaved, for instance, by neutrophil-released proteases [10–13]. Thus, to test whether in addition to the lower levels of inhibitors found in PsA compared with RA synovitis there was also an increased IL-36 activation, we investigated the expression of neutrophil-related genes and Neutrophil elastase and Cathepsin G, the two serine proteinases involved in the maturation of IL-36α [12]. Interestingly, PsA ST was characterized by the up-regulation of neutrophil-related genes such as *CCR3*, *LRG1* or *CXCR1* (Fig. 2A). Furthermore, Neutrophil elastase and Cathepsin G were significantly more expressed in PsA synovium compared with RA (Fig. 2B–E). However, Cathepsin S, the major activator of IL-36γ in the skin [13], is expressed at the same level in PsA and RA ST (supplementary Fig. S2A and B, available at *Rheumatology* online). Altogether, these findings suggest that PsA synovium is characterized by a strong neutrophil signature, which, plausibly, favours a higher activation of the IL-36 axis.

### IL-36 cytokines and their antagonists are differentially expressed depending on the synovial histological pathotypes in PsA and RA

**Fig. 3:**
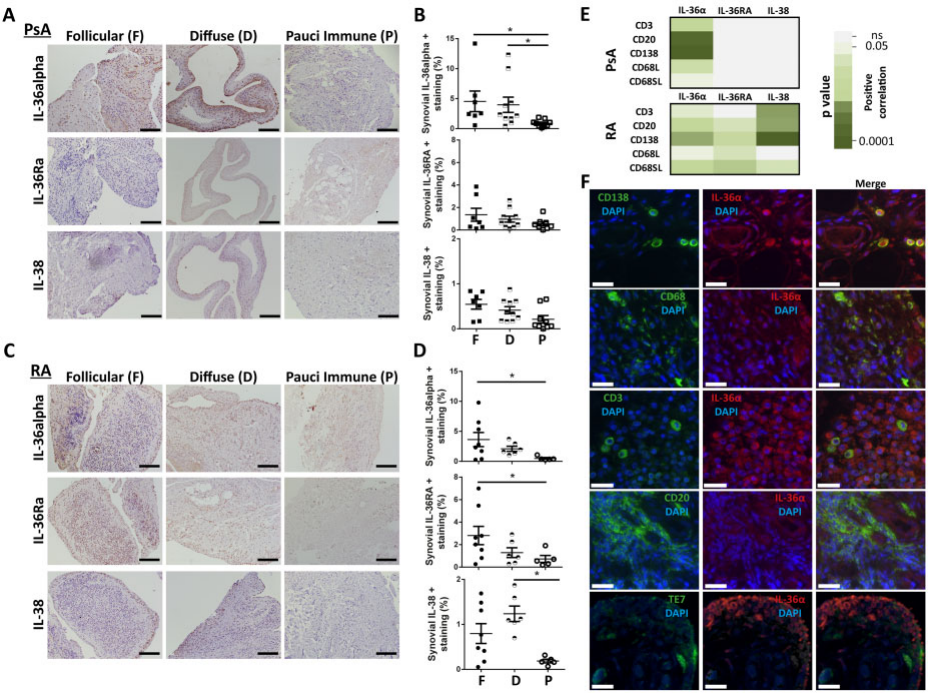
Association between IL-36α, IL-36RA and IL-38 expression and the synovial histomorphology in early DMARDs-naïve PsA and RA patients (**A**, **C**) Sections of PsA and RA synovium were stained for IL-36α, IL-36RA and IL-38. In RA synovium, three main histological pathotypes have been described previously: follicular (F), characterized by the presence of B/T cells frequently forming highly organized ELS, plasma cells and macrophages in the sublining; diffuse (D), marked by a predominant infiltration of CD68+ macrophages without distinctly organized follicular structures; and pauci-immune (P), defined by a scant immune cells infiltrate and a fibroblast-rich stroma. Representative images are shown for each pathotypes: F, D and P. Scale bar = 100 μm. (**B**, **D**) Digital image analysis was performed on PsA (*n*=27) and RA (*n*=19) synovium sections for each pathotypes (F, D and P). IL-36α+, IL-36RA+ and IL-38+ surfaces were determined using ImageJ software and are presented as % of the synovium surface. *P < 0.05 as assessed by Kruskal–Wallis with Dunn’s post-test. (**E**) Correlations between IL-36α, IL-36RA, IL-38 and inflammatory cells markers (CD3-T cells; CD20-B cells; CD138-plasma cells; CD68L-macrophages of the synovium lining layer; CD68SL-macrophages of the sublining layer) are shown. Positive significant correlations at the tissue protein expression level are presented in green. The green scale indicates the *P*-values, calculated by Spearman’s bivariate correlation analysis. Non-significant correlations are presented in white. (**F**) Double immunostaining of IL-36α with CD138, CD68, CD3, CD20 and TE7 in the synovium of PsA patients. IL-36α is shown in red; CD3, CD20, CD138, CD68 or TE7 are shown in green. Nuclei were counterstained with 40, DAPI (blue). Co-localizations of IL-36 with the cellular markers appear in yellow/light green (Merge). Representative images are shown. Scale bar = 20 μm. DAPI: 6-diamidino-2-phenylindole; ELS: ectopic lymphoid structures.

PsA synovitis shows a similar tissue heterogeneity as observed in RA, and the three previously described pathotypes (follicular, diffuse and pauci-immune) can be detected (supplementary Fig. S3A, available at *Rheumatology* online) [32]. Interestingly, we noted that the IL-36 cytokines and their endogenous inhibitors displayed different profiles of expression depending on both the histological features of the ST and the type of disease. In PsA, the higher expression of agonists in the follicular and diffuse pathotypes was not matched by an adequate up-regulation of the antagonists (Fig. 3A and B and supplementary Fig. S3B–E, available at *Rheumatology* online). Conversely, in RA-follicular synovitis, the higher expression of IL-36 agonists was accompanied by a significantly greater availability of the antagonist IL-36RA (Fig. 3C and D). As represented in Fig. 3E and supplementary Fig. S3F, available at *Rheumatology* online, in PsA, only agonists expression levels positively correlated with the degree of synovial infiltration of CD3+ T cells, CD20+ B cells, CD138+ plasma cells and CD68+ macrophages. In contrast, in RA, both the IL-36 agonist and inhibitors positively and concomitantly correlated with the immune cells infiltrate. These observations further suggest that, in PsA ST, the IL-36 cytokines, inadequately counterbalanced by their endogenous inhibitors, drive the local inflammation.

As previously reported [28], we confirmed that within PsA ST, CD138+ plasma cells are a source of IL-36α and we demonstrated for the first time that, as described in RA [22], CD68+ macrophages in PsA also express IL-36α; to a lesser extent, IL-36α is also expressed by CD3+ T cells, CD20+ B cells and TE7+ fibroblasts (Fig. 3F).

### PsA patients are characterized by higher co-expression of IL-36α/IL-36R in the ST and lower levels of circulating of IL-36α

**Fig. 4:**
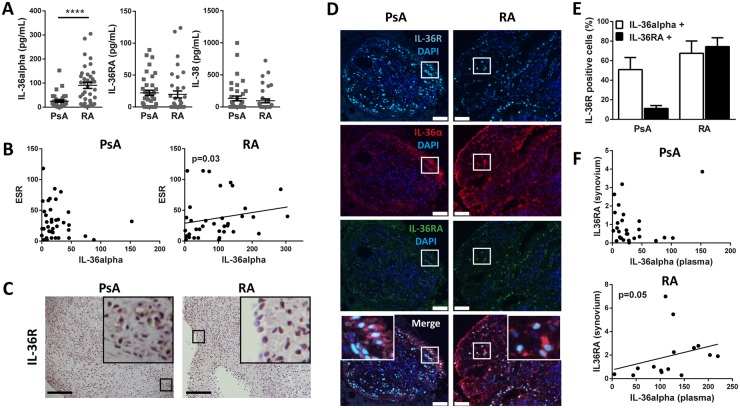
Relationship between synovial and systemic expression of IL-36α and their antagonists (**A**) IL-36α, IL-36RA and IL-38 protein levels (pg/ml), as assessed by ELISA in plasma of early DMARDs-naïve PsA (*n*=38) and RA (*n*=38) patients. The results are shown as individual values, mean (s.e.m). ****P < 0.0001 as assessed by unpaired Student’s *t* test. (**B**) Correlation between the ESR and IL-36α expression in the plasma of PsA and RA patients is represented. The *P*-value is indicated, as assessed by Spearman’s bivariate correlation analysis. (**C**) Sections of human PsA and RA synovium were stained for IL-36R (IL-1Rrp2-specific chain). Representative images are shown. Scale bar = 100 μm. Enlarged images correspond to the respective boxed areas. Digital image analysis was performed and is shown in supplementary Fig. S3, available at *Rheumatology* online. (**D**) Triple immunostaining of IL-36α with IL-36R and IL-36RA in the synovium of PsA and RA patients. IL-36α is shown in red, IL-36R in light blue and IL-36RA in green. Nuclei were counterstained with DAPI and are shown in blue. Representative images are shown. Enlarged images correspond to the respective white boxed areas. Scale bar = 100 μm. (**E**) Percentage of synovial IL-36R+ cells from early PsA and RA patients positive for IL-36α or IL-36RA. Cells double-positive for IL-36R and IL-36α (in white) or IL-36R and IL-36RA (in black) were manually counted using the cell count plugin of ImageJ. (**F**) Correlation between IL-36α expression in the plasma and IL-36RA in the synovium is shown. *P*-values (shown) were calculated by Spearman’s bivariate correlation analysis. DAPI: 6-diamidino-2-phenylindole.

We next quantified the circulating levels of IL-36α in plasma of PsA and RA patients. Circulating plasma concentrations of IL-36α were significantly higher in RA patients compared with PsA, whereas no differences were observed for the antagonists IL-36RA and IL-38 (Fig. 4A). In RA, but not PsA, plasma IL-36α also positively correlated with the systemic inflammatory burden measured by ESR (Fig. 4B).

Since the pro-inflammatory activity of the IL-36 axis is mediated by the binding of the IL-36 agonists to the receptor IL-36R, we wondered whether the lower levels of circulating IL-36α observed in PsA patients could be explained by a more effective ligand/receptor binding within the ST. As shown in Fig. 4C and supplementary Fig. S4, available at *Rheumatology* online, there were no significant differences between PsA and RA in the synovial expression of the IL-36R. However, while in PsA the agonist IL-36α was mainly co-expressed with its receptor, suggesting an active ligand/receptor binding, in RA 75% of IL-36R co-localized with the antagonist IL-36RA, which inhibits the IL-36 downstream signalling by blocking IL-36 binding to the receptor (Fig. 4D and E). Furthermore, in RA but not in PsA, the plasma level of IL-36α positively correlated with the synovial expression of the antagonist IL-36RA (Fig. 4F).

### High synovial IL-36α expression predicts inadequate response to DMARDs in PsA and is not abrogated by MTX and sulfasalazine

**Fig. 5:**
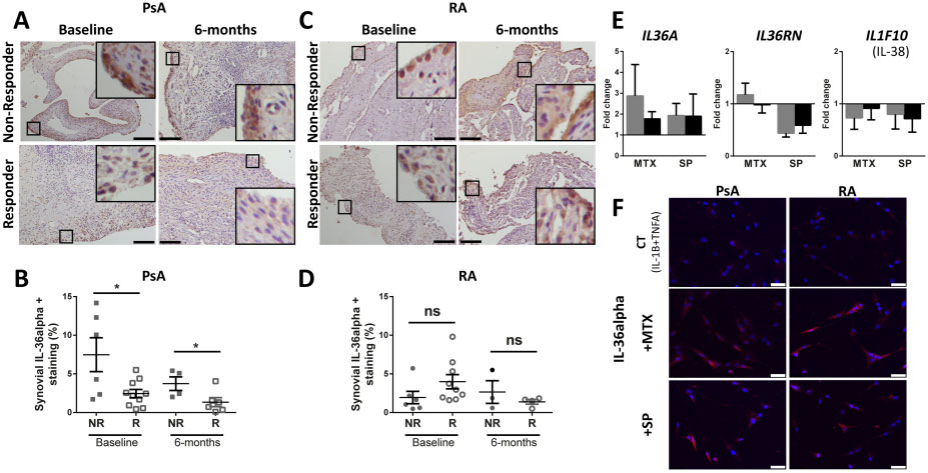
IL-36α is highly expressed in the synovium of DMARDs non-responders PsA patients (**A**, **C**) Matched baseline and post-DMARDs (6 months) synovial tissue sections of R and NR PsA or RA patients were stained for IL-36α. Representative images are shown. Scale bar = 100 μm. Enlarged images correspond to the respective boxed areas. (**B**, **D**) Digital image analysis was performed on PsA (NR: *n* = 6 at baseline and *n* = 4 at 6 months; R: *n* = 10 at baseline and *n* = 6 at 6 months) and RA (NR: *n* = 6 at baseline and *n* = 3 at 6 months; R: *n* = 9 at baseline and *n* = 4 at 6 months) synovium sections. *P < 0.05 as assessed by Mann–Whitney *U* test, ns. (**E**) PsA (*n* = 7, in grey) or RA (*n* = 8, in black) FLS were stimulated with IL-1β and TNF-α (25 ng/ml each) with either MTX or SP (1 mM) and RNA expression of IL-36A, IL-36RN and IL-1F10 (*IL-38* gene) was assessed by RT-quantitative PCR after 24 h of stimulation. (**F**) Immunocytochemistry staining of IL-36α in FLS stimulated with IL-1β and TNF-α (25 ng/ml each) together with 1 mM of MTX or SP for 36 h. IL-36α is shown in red, nuclei are stained with DAPI and represented in blue. DAPI: 4′,6-Diamidino-2-phenylindole; FLS: Fibroblast-Like Synoviocytes; NR: non-responders; ns: non-significant; R: responders; SP: sulfapyridine.

In keeping with our hypothesis that IL-36 cytokines play a pathogenic role in PsA, we hypothesized that differential synovial expression of the agonist IL-36α would correlate with the clinical response to standard anti-rheumatic treatment. We demonstrated that PsA (but not RA) inadequate responders to 6 months’ therapy with conventional synthetic (cs) DMARDs, including MTX and sulfasalazine, were characterized by significantly higher levels of synovial IL-36α expression both at baseline (pre-treatment) and at 6 months after starting the treatment (Fig. 5A–D). However, no significant difference was observed, either for the other agonists IL-36β and IL-36γ, or for the antagonists IL-36RA and IL-38 (supplementary Fig. S5A, available at *Rheumatology* online). To test whether csDMARDs were able to down-regulate the pro-inflammatory IL-36 agonists, we showed that MTX or SP (an active metabolite of sulfasalazine) treatment of *in vitro*-cultured primary RA/PsA-FLS challenged with IL-1β and TNF-α appeared to cause an increase of IL-36α transcript rather than a decrease, whereas the treatment did not increase classical pro-inflammatory cytokines such as IL-1β, TNF-α or IL-8 (Fig. 5E and supplementary Fig. S5B, available at *Rheumatology* online). MTX/SP-induced IL-36α expression by FLS was also observed at the protein level by immunofluorescence (Fig. 5F). At the same time, genes encoding the inhibitors IL-36RA and IL-38 were reduced by MTX and SP treatment (Fig. 5E), therefore promoting an imbalance between agonists and antagonists in favour of pro-IL-36 pathway activation. DMSO control stimulation is provided in supplementary Fig. S5C and D, available at *Rheumatology* online.

### Primary PsA-derived FLS are more responsive to IL-36 stimulation

**Fig. 6:**
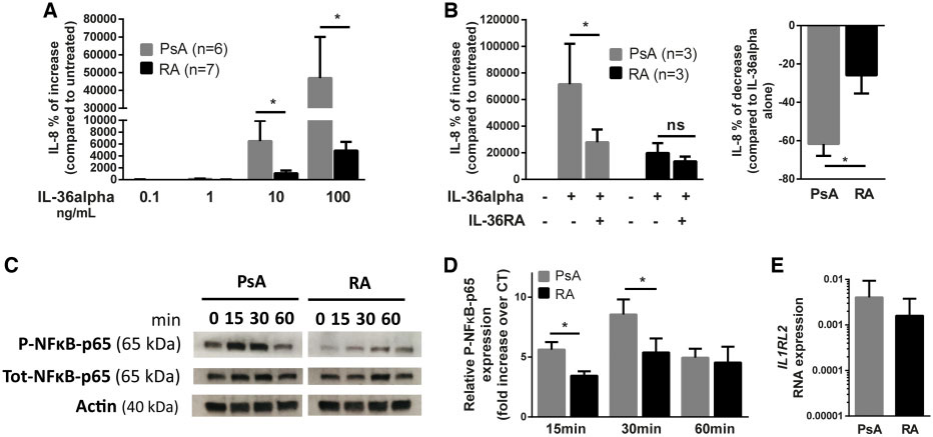
Primary FLS from PsA patients are more sensitive to IL-36 stimulation (**A**) PsA (*n*=6 individual patients) and RA (*n*=7 individual patients) FLS were stimulated with increasing doses of rhIL-36α (0.1–100 ng/ml) for 48 h. IL-8 concentration in the supernatants was determined by ELISA; the percentage of increase compared with supernatants of untreated cells is shown. *P < 0.05 as assessed by Kruskal–Wallis with Dunn’s post-test. (**B**) IL-8 was measured in the supernatants of PsA (*n*=3) and RA (*n*=3) FLS stimulated with rhIL-36α for 48 h with or without pre-treatment (1 h) with 100 ng/ml of rhIL-36RA. The percentage of increase compared with supernatants of untreated cells (left panel) and the percentage of decrease compared with cells stimulated with IL-36α alone (right panel) are shown. *P < 0.05 as assessed by Kruskal–Wallis with Dunn’s post-test (left panel) or the Mann–Whitney *U* test (right panel). (**C**) Protein expression of total and phosphorylated NF-κB in PsA- and RA-FLS stimulated with 100 ng/ml of rhIL-36α for 15, 30 or 60 min. Actin is used as loading control. A representative blot of three independent experiments is shown. (**D**) Quantification of western blot signal for phosphorylated NF-κB using ImageJ software. Signals were normalized to the Actin signal and the fold increase is calculated relative to the untreated condition. Results are presented as mean (s.e.m). *P < 0.05 as assessed by Mann–Whitney *U* test for each timepoint. (**E**) IL-1RL2 (gene encoding IL-36Rrp2) expression was assessed in PsA- and RA-FLS by RT-quantitative PCR. FLS: Fibroblast-Like Synoviocytes; NF-κB: nuclear factor κB.

We next investigated the effects of IL-36α on primary FLS, its main cellular target within the synovium [23, 24]. IL-8 secretion, typically induced in FLS upon an inflammatory stimulus, was significantly increased in PsA-FLS compared with RA-FLS following IL-36α stimulation (Fig. 6A). IL-6 secretion by FLS, instead, was not different between PsA and RA upon IL-36α stimulation (supplementary Fig. S6A, available at *Rheumatology* online). Moreover, the differential expression of IL-8 by PsA- and RA-FLS was lost following TNF-α stimulation, suggesting that IL-36α has a disease-specific effect on FLS response (supplementary Fig. S6B, available at *Rheumatology* online). Since we observed a reduced availability of IL-36 antagonists in PsA ST (Fig. 1B–D), we hypothesize that adding exogenous IL-36RA could inhibit IL-36α-induced IL-8 secretion by RA/PsA-FLS. As shown in Fig. 6B, IL-36RA addition was able to significantly down-regulate the production of IL-8 by PsA-FLS previously stimulated with IL-36α, while no meaningful differences were observed in RA-FLS. IL-36α activated more efficiently the NF-κB pathway in PsA-FLS *vs* RA-FLS, as demonstrated by the increased phosphorylation of p65 at 15 and 30 min after stimulation (Fig. 6C and D), suggesting that intracellular pro-inflammatory signalling downstream of IL-36 is more active in PsA than in RA. The level of the transcript encoding the specific receptor of IL-36, *IL-1Rrp2*, was comparable between PsA and RA ST (Fig. 6E); thus, the significantly higher expression of IL-36α-induced IL-8 in PsA is unlikely to be due to greater availability of IL-36 receptor.

## Discussion

Several studies have already highlighted the key role played by IL-36 agonists [34], especially IL-36α [17, 18], in the pathogenesis of psoriatic skin disease. Here, we have analysed the state of the synovial IL-36 axis in the synovium of early treatment-naïve PsA patients.

As previously demonstrated by Frey and colleagues [28], we also confirm that IL-36α is expressed at the same level in PsA and RA synovium. Similar to RA, in PsA IL-36α is mainly produced by plasma cells and macrophages. However, an important novel finding of our study is that PsA synovium is characterized by deficient expression of the antagonists IL-36RA and IL-38 in comparison with RA. Accordingly, PsA synovitis shows a pro-inflammatory IL-36 agonist/antagonist ratio analogous to that found in psoriatic lesional skin [22], in which the IL-36 axis has recently been recognized as having an important pathogenic role. Unfortunately, the study design of the pathobiology of early arthritis cohort focussed on the synovium and did not include skin biopsy in the early treatment-naïve PsA patients. Therefore, further investigations on matched skin–synovium tissues would be required to confirm this hypothesis in the two disease compartments.

Importantly, however, our results indicate that given the lower expression of antagonists within PsA ST, IL-36α is more likely to bind its receptor and activate the downstream pro-inflammatory cascade. Conversely, in RA synovium, the antagonist IL-36RA, which is significantly expressed at higher level, can competitively bind the IL-36R, and, due to its higher affinity, inhibits the IL-36 pathway activation. It is plausible that increased IL-36 receptor blockade by IL-36RA may explain higher circulating unbound IL-36α plasma levels in RA.

It has been demonstrated that IL-36 cytokines must be processed to gain full pro-inflammatory activity [9]. Since the available antibodies detecting IL-36α target both the full and the truncated isoform, we could not prove by immunostaining that IL-36 was fully activated in PsA ST. We have, however, demonstrated that PsA synovium is defined by a strong neutrophil gene expression signature and that both Neutrophil elastase and Cathepsin G, the proteases involved in the maturation of IL-36α [12], are significantly more expressed in PsA *vs* RA. Additionally, since unprocessed IL-36α maintains the capability of driving psoriasis-like inflammation in mice [17], it is conceivable that PsA synovium represents a conducive tissue for the pro-inflammatory activity of both IL-36α isoforms.

IL-36 agonists have been proven to have pro-inflammatory effects on cells of the skin and to be involved in the development of skin changes in psoriasis [35]. Consistently, we confirmed the importance of the IL-36 cytokines in PsA by showing that primary FLS derived from inflamed psoriatic joints are more responsive to IL-36α stimulation, as demonstrated by the elevated production of IL-8 and stronger NF-κB activation in PsA-FLS compared with RA-FLS. Interestingly, this disease-dependent response is specific for IL-36; TNF-α stimulation, in fact, causes a comparable production of pro-inflammatory molecules by both PsA- and RA-FLS. Moreover, IL-36RA treatment was more effective in down-regulating the production of IL-8 by PsA-FLS compared with RA-FLS. We hypothesized that RA synovial cells can operate an autocrine negative feedback on the activation of the IL-36 pathway, in keeping with the lower ratio of agonists/antagonists observed in the whole tissue. Conversely in PsA, similar to skin psoriasis, this homeostatic regulation is lost, but adding IL-36 inhibitors exogenously could restore the down-regulation of the inflammatory cascade. These observations may have considerable translational applications in PsA.

In fact, blocking the IL-36 receptor has been already been shown to be a successful strategy in pre-clinical models of psoriasis [18], and clinical trials are ongoing to validate the safety and efficacy of this therapeutic approach in human [36, 37]. In addition, recent studies have outlined the ability of neutrophil proteases inhibitors to reduce IL-36-driven inflammation, suggesting their potential therapeutic role in the context of psoriasis, and strengthening further the importance of the IL-36 pathway in sustaining psoriasis-related disease [38, 39].

The inadequate response of PsA patients to csDMARDs represents a significant unmet clinical need. The introduction of biologic agents targeting TNF-α and the IL-17/IL-23 axis has improved outcomes for PsA patients; however, the rate of non-responders remains unacceptably high [2]. Therefore, blocking the IL-36 pathway in PsA may be a new powerful tool for ameliorating disease in difficult-to-treat patients. We have demonstrated that early in the disease, prior to treatment intervention, higher synovial expression of IL-36α predicts poor response to csDMARDs. More importantly, we have also shown that IL-36α remains significantly higher in the ST of non-responder patients even after receiving the treatment, suggesting that the persistent expression of this axis drives chronic inflammation locally and represents an active pathway that could potentially be targeted therapeutically. We observed an inability of the most commonly used csDMARDs to antagonize the IL-36 pathway using *in vitro* experiments in which treating PsA-derived synovial fibroblasts with MTX and SP triggered the production of IL-36 agonists while down-regulating the expression of the antagonists and other pro-inflammatory cytokines such as IL-1β and TNF-α. Overall, these data suggest that, in patients who are inadequate responders to treatment, the IL-36 axis becomes one of the most relevant active inflammatory pathways.

The advantages of a personalized therapeutic approach to patients with arthritis have been lately recognized [40], and the assessment of the histopathology of the ST is one of the most promising candidate approaches. Here, we provide important information about the relationship that exists between the expression of the IL-36 family members and the histological features of the synovitis in different forms of inflammatory arthropathy, which might be exploitable for refining the therapeutic targeting of active pathways in specific synovial pathotypes.

In conclusion, we have here demonstrated that the impaired ratio between IL-36 agonists and antagonists is critical to drive the inflammation within the ST of PsA patients and provided the first evidence that the exogenous replacement of IL-36 antagonists may represent a novel promising therapeutic approach in PsA.

## Supplementary Material

kez358_Supplementary_Data
