# Supplementary material for: Interleukin-36 family dysregulation drives joint inflammation and therapy response in psoriatic arthritis
Source: Rheumatology (Oxford). 2019 Sep 3;59(4):828–38. doi: 10.1093/rheumatology/kez358 (PMC7188345; doi:10.1093/rheumatology/kez358)
Supplement: kez358_Supplementary_Data [file kez358_supplementary_data.zip › kez358-suppl_data/rhe-19-0527-File008.docx]

SUPPLEMENTARY MATERIAL

Supplementary Table S1 Demographic and clinical characteristics of the PsA and RA patients. p values were determined by either Mann–Whitney U-test (<30), unpaired Student’s t-test (>30) for comparison of mean values, and by Fisher's exact test (<30) or chi-square test (>30) for evaluation of significant differences in proportions. RNA-Seq = RNA-Sequencing; IHC = Immunohistochemistry; ELISA = Enzyme-Linked Immunosorbent Assay; SD = Standard Deviation; DAS28 = Disease Activity Score in 28 joints; ESR = Erythrocyte Sedimentation Rate; CRP = C-Reactive Protein; CCP = Cyclic Citrullinated Peptide; RF = Rheumatoid Factor.

**Supplementary Table S2 Details of antibodies (name, supplier, specie and clone) and concentrations used for immunostaining.**

**Supplementary figure legends**

Supplementary Figure S1 Expression of IL-36β and IL-36γ in synovium from early DMARDs-naïve PsA and RA patients. (A-B) Sections of human PsA and RA synovium were stained for IL-36β (A, left panels) and IL-36γ (B, left panels). Representative images are shown. Scale bar = 100μm. Enlarged images correspond to the respective boxed areas. Digital image analysis was performed on all PsA (n=27) and RA (n=19) synovium sections. IL-36β+ (A, right panel) and IL-36γ (B, right panel) surfaces were determined using Image J software (NIH, Bethesda, MD, USA) and are presented as % of the synovium surface. (C) Ratios between agonist (IL-36 β and γ) and antagonists (IL-36RA and IL-38) expressions are shown. (A-C) Results are presented as mean ± SEM. * = p < 0.05, as assessed by Mann–Whitney U-test.

**Supplementary Figure S2** **Cathepsin S is similarly expressed in PsA and RA synovium.** (A) Sections of human PsA and RA synovium were stained for Cathepsin S. Representative images are shown. Scale bar = 100μm. Enlarged images correspond to the respective boxed areas. (B) Digital image analysis was performed on all PsA (n=27) and RA (n=19) synovium sections. Cathepsin S positive staining surfaces were determined using Image J software and are presented as % of the synovium surface. Results are presented as mean ± SEM.

**Supplementary Figure S3 Diverse histomorphological features of synovitis in PsA and association between IL-36β and IL-36γ and the synovial histomorphology in early DMARDs-naïve PsA and RA patients.** (A) Sections from human PsA synovium were stained for CD3 (T cells), CD20 (B cells), CD68 (macrophages) and CD138 (plasma cells). Prototypical examples of each pathotypes are shown: Follicular -F-, rich in B/T cells aggregates; Diffuse -D-, characterized by a predominant macrophage component; and Pauci Immune -P-, rich in synovial fibroblasts but with a scant immune-cells infiltration; Scale bar = 100μm. (B, D) Sections of PsA and RA synovium were stained for IL-36β and IL-36γ. Representative images are shown for each pathotypes: F, D and P. Scale bar = 100μm. (C, E) Digital image analysis was performed on PsA (n=27) and RA (n=19) synovium sections for each pathotypes (F, D and P). IL-36β+ and IL-36γ+ surfaces were determined using Image J software (NIH, Bethesda, MD, USA) and are presented as % of the synovium surface. * = p < 0.05, ** = p < 0.01 as assessed by Kruskal–Wallis with Dunn's post-test. (F) Correlations between IL-36β and IL-36γ and inflammatory cells markers (CD3-T cells; CD20-B cells; CD138-plasma cells; CD68L-macrophages of the synovium lining layer; CD68SL-macrophages of the sublining layer) are shown. Positive significant correlations at the tissue protein expression level are presented in green. The green scale indicates the p values, calculated by Spearman’s bivariate correlation analysis.

**Supplementary Figure S4 Quantification of IL-36R expression in PsA and RA synovium**. Sections of human PsA and RA synovium were stained for IL-36R (IL-1Rrp2 specific chain) (Figure 4) and digital image analysis was performed on all RA (n=19) and PsA (n=27) synovium sections. IL-36R+ surfaces were determined using Image J software (NIH, Bethesda, MD, USA) and are presented as % of the synovium surface.

**Supplementary Figure S5 IL-36α, but not IL-36β, γ, IL-36RA or IL-38, is highly expressed in the synovium of DMARDs non-responders PsA patients.** (A) Matched baseline and post-DMARDs (6-months) synovial tissue sections of responders (R) and non-responders (NR) PsA patients were stained for IL-36β, γ, IL-36RA and IL-38. Digital image analysis was performed on PsA (NR: n=6 at baseline and n=4 at 6 months; R: n=10 at baseline and n=8 at 6 months) synovium sections. (B) PsA (n=7, in grey) or RA (n=7, in black) Fibroblast-Like Synoviocytes (FLS) were stimulated with IL-1β and TNFα (25ng/mL each) with either Methotrexate (MTX) or Sulfapyridine (SP) (1mM) and RNA expression of IL1B, TNF and IL8 was assessed by RT-qPCR after 24 hours of stimulation. (C) FLS (n=5) were stimulated with IL-1β and TNFα (25ng/mL each) alone or together with DMSO control (quantity equivalent to condition with MTX or SP at 1mM) and RNA expression of IL36A, IL36RA or IL1F10 (IL-38 gene) was assessed by RT-qPCR after 24 hours of stimulation. (D) Immunocytochemistry staining of IL-36α in PsA-FLS stimulated with IL-1β and TNFα (25ng/mL each) alone or together with DMSO control, MTX or SP for 36 hours. IL-36α is shown in red, nuclei are stained with DAPI and represented in blue.

Supplementary Figure S6 Primary Fibroblast-Like Synoviocytes (FLS) from PsA patients produces more IL-8 compared to RA-FLS after IL-36 stimulation, but not after TNF stimulation. (A) PsA (n=7 individual patients) and RA (n=7 individual patients) FLS were stimulated with increasing doses of rhIL-36α (0.1 to 100ng/mL) for 48 hours. IL-6 concentration in the supernatants was determined by Enzyme linked Immunosorbent Assay (ELISA); the percentage of increase compared to supernatants of untreated cells is shown. (B) IL-8 (left panel) and IL-6 (right panel) were measured in the supernatants of PsA (n=3) and RA (n=3) FLS stimulated with rhIL-36α or rhTNFα at the indicated concentration for 48 hours. * = p < 0.05 as assessed by Kruskal–Wallis with Dunn's post-test.

Supplementary Table S1 Demographic and clinical characteristics of the PsA and RA patients. p values were determined by either Mann–Whitney U-test (<30), unpaired Student’s t-test (>30) for comparison of mean values, and by Fisher's exact test (<30) or chi-square test (>30) for evaluation of significant differences in proportions. RNA-Seq = RNA-Sequencing; IHC = Immunohistochemistry; ELISA = Enzyme-Linked Immunosorbent Assay; SD = Standard Deviation; DAS28 = Disease Activity Score in 28 joints; ESR = Erythrocyte Sedimentation Rate; CRP = C-Reactive Protein; CCP = Cyclic Citrullinated Peptide; RF = Rheumatoid Factor.

Supplementary Table S2 Details of antibodies (name, supplier, specie and clone) and concentrations used for immunostaining.
