# Supplementary figures and images for: Interleukin-36 family dysregulation drives joint inflammation and therapy response in psoriatic arthritis
Source: Rheumatology (Oxford). 2019 Sep 3;59(4):828–38. doi: 10.1093/rheumatology/kez358 (PMC7188345; doi:10.1093/rheumatology/kez358)

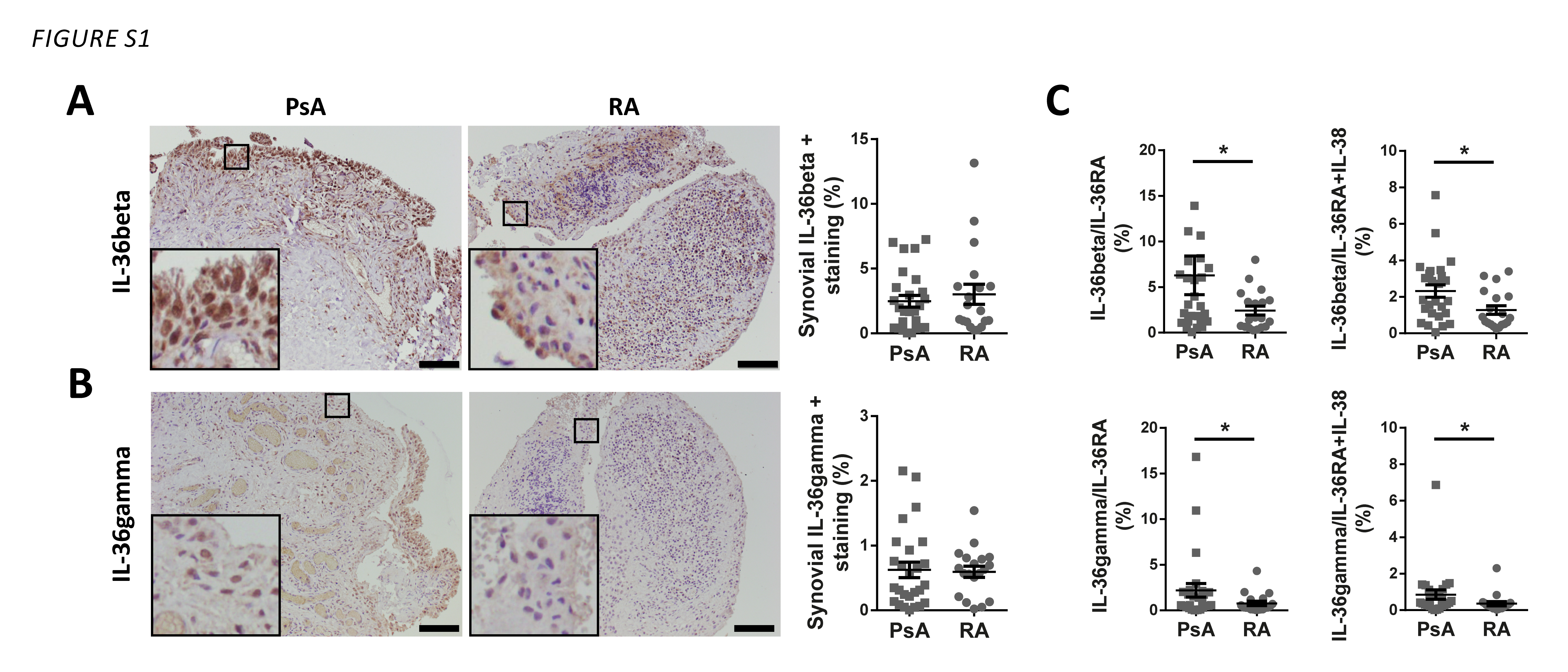

Supplement: kez358_Supplementary_Data [file kez358_supplementary_data.zip › kez358-suppl_data/rhe-19-0527-File009.tif]

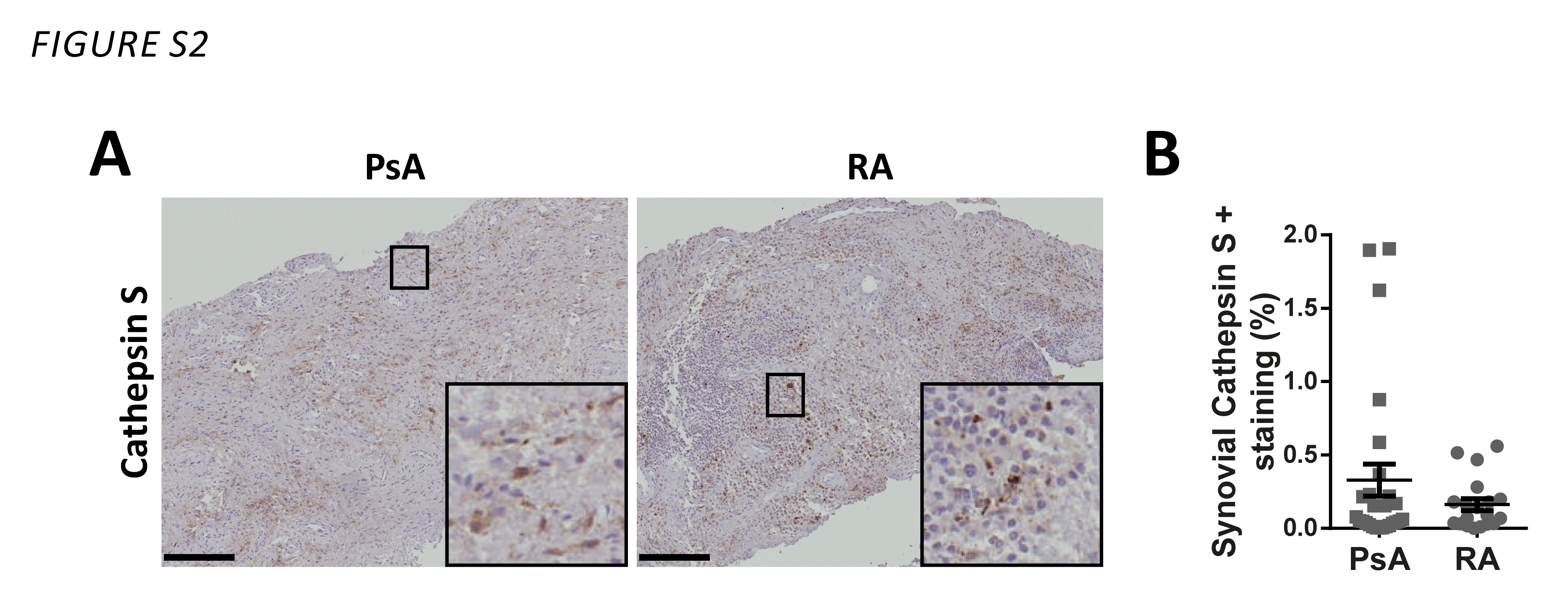

Supplement: kez358_Supplementary_Data [file kez358_supplementary_data.zip › kez358-suppl_data/rhe-19-0527-File010.tif]

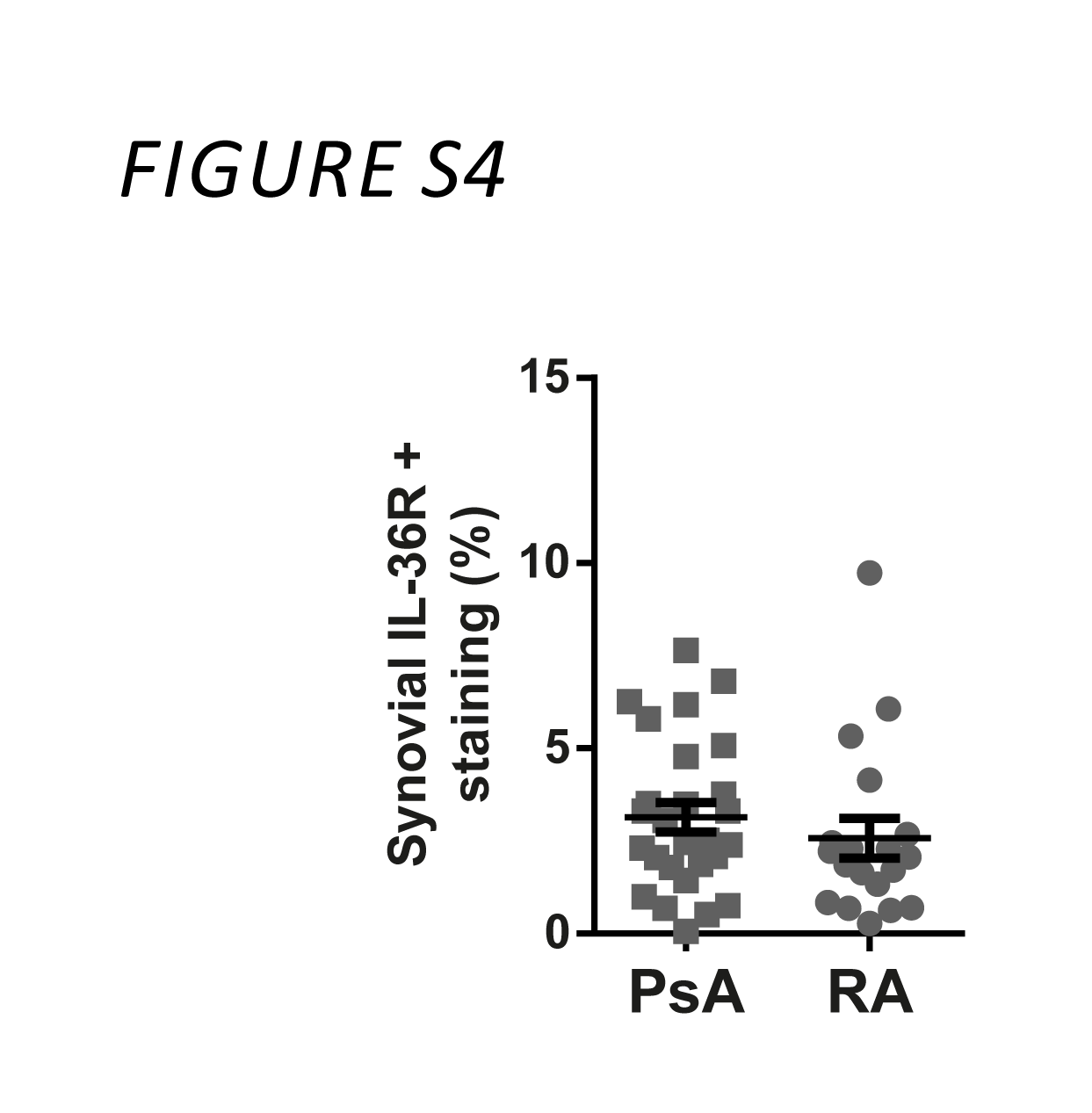

Supplement: kez358_Supplementary_Data [file kez358_supplementary_data.zip › kez358-suppl_data/rhe-19-0527-File012.tif]

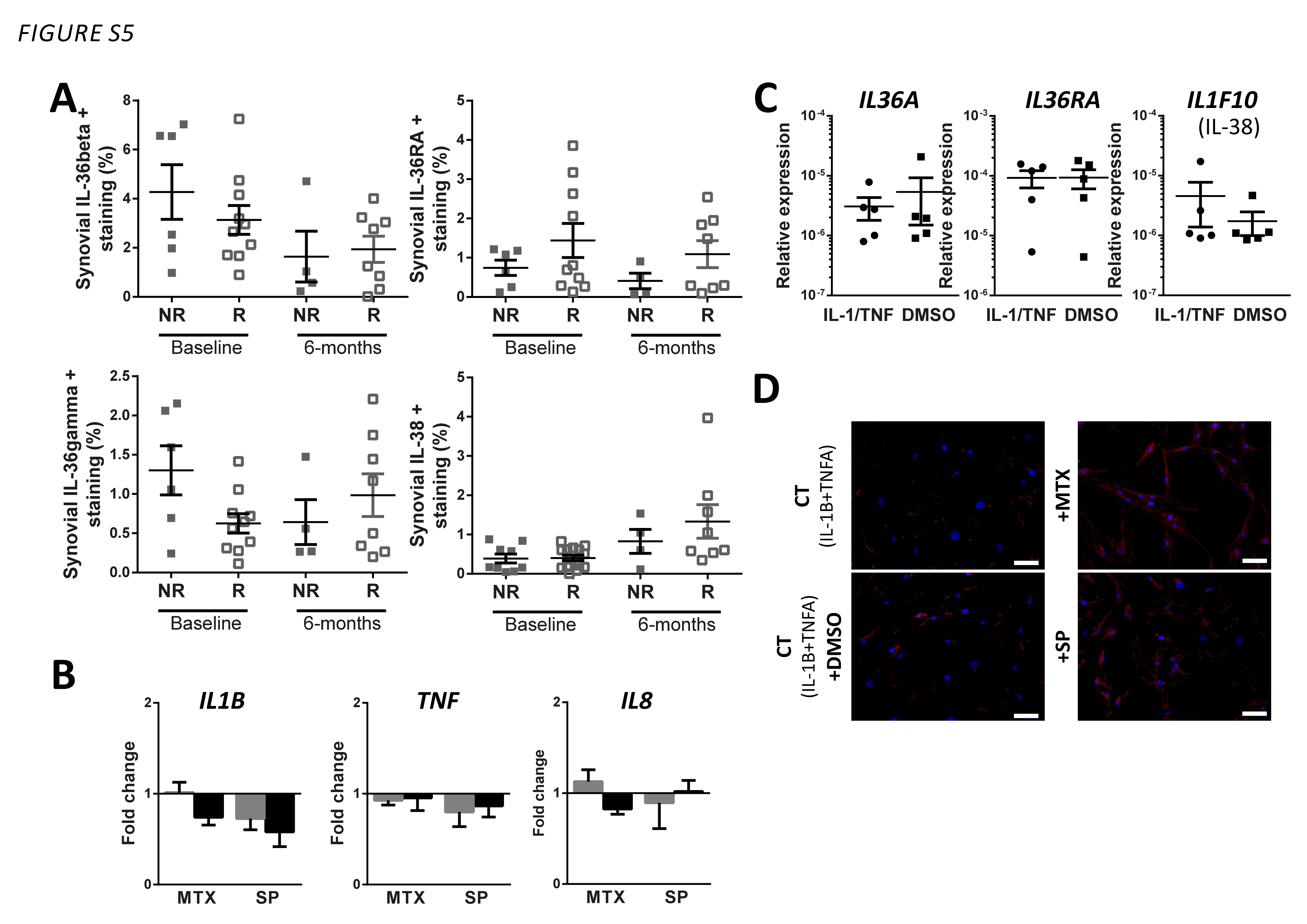

Supplement: kez358_Supplementary_Data [file kez358_supplementary_data.zip › kez358-suppl_data/rhe-19-0527-File013.tif]

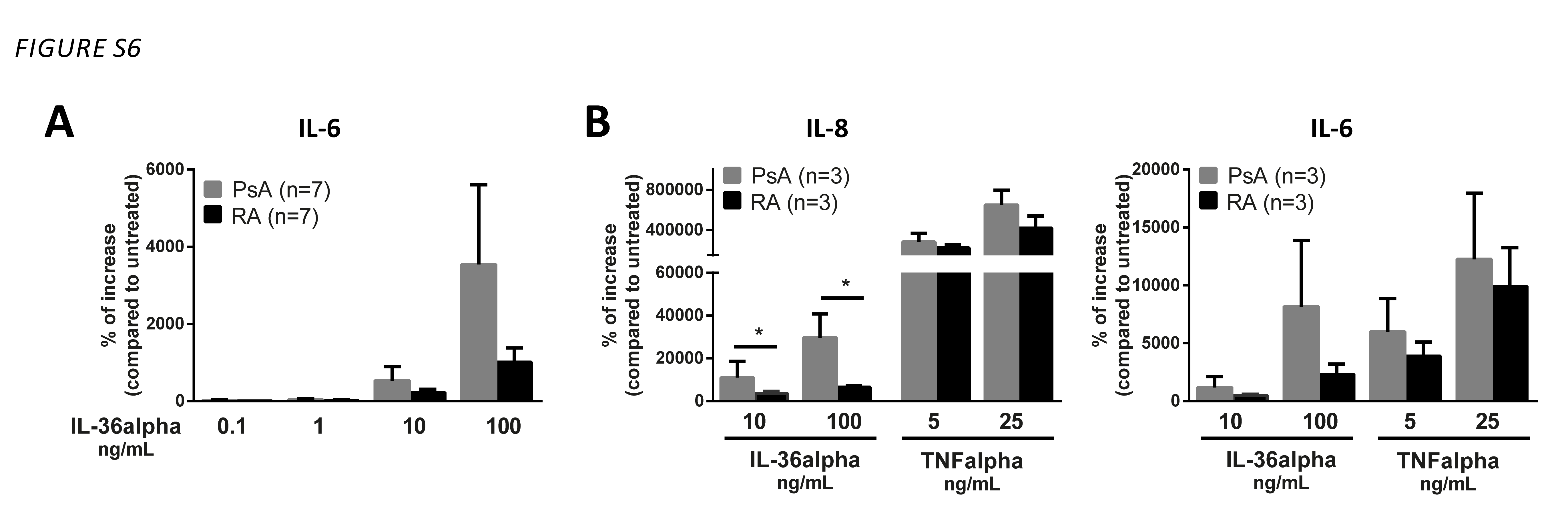

Supplement: kez358_Supplementary_Data [file kez358_supplementary_data.zip › kez358-suppl_data/rhe-19-0527-File014.tif]
